# Supplementary material for: Cost-effectiveness of esketamine versus alternative treatment strategies for treatment-resistant depression in Hong Kong: A multi-armed modeling study
Source: PLoS Med. 2026 Apr 16;23(4):e1005047. doi: 10.1371/journal.pmed.1005047 (PMC13120699; doi:10.1371/journal.pmed.1005047)
Supplement: S1 Appendix — Table A. Model input parameters. Table B. Scenario analysis of per patient cost, QALYs and ICER in each comparative arm. Fig A. Schema of retrospective cohort analysis for costs of healthcare resource utilization. Fig B. Deterministic Sensitivity Analysis (DSA) results for ICER of esketamine arm across key model parameters. (DOCX) [file pmed.1005047.s001.docx]

**S1 Appendix. Supplemental Online Content**

**Supporting Methods**

**Table A.** Model input parameters

**Table B.** Scenario analysis of per patient cost, QALYs and ICER in each comparative arm

**Fig A.** Schema of retrospective cohort analysis for costs of healthcare resource utilisation

Fig B. Deterministic Sensitivity Analysis (DSA) Results for ICER of Esketamine Arm Across Key Model Parameters

**Supporting Methods**

- 1. **Demographics and Mortality**

In the EMR database, we extracted all patients aged above 18 with new depression diagnoses between 2014 and 2016 and ascertained to develop TRD as our reference cohort, resulting in a final reference cohort of 480 individuals. The mean age was 49.5 (SD: 17.2) of which 74.6% were female. Further details of this cohort and TRD ascertainment were published previously^3,23^. Time-dependent mortality rates varied yearly were adapted from our previous burden projection model specific to the TRD population^23^.

- 1. **Clinical Effectiveness**

As the head-to-head comparative literature is limited, a target systematic review was conducted to identify meta-analyses and clinical trials comparing the efficacy of esketamine and other treatment choices to oral AD monotherapy, which acted as a common referent for indirect comparison. Each comparison arm captures the aggregated health effects and costs of a range of medications or interventions grouped under the same strategy, whilst the choice of included therapies per arm depended on the availability of pooled evidence in the search. To improve comparability, we prioritised literature that studied selective serotonin reuptake inhibitors (SSRIs) or serotonin-norepinephrine reuptake inhibitors (SNRIs) as the oral antidepressant monotherapy control arm. All effectiveness parameters represent the treatment effects of each strategy compared with oral AD monotherapy. We referenced remission, response, and relapse rates from the STAR*D study^1^. We assumed the clinical effectiveness of the third-line, fourth-line and fifth-line treatments in the base-case to be equivalent to those of the level 2 (oral AD monotherapies only), level 3 and level 4 treatments evaluated in STAR*D respectively, whilst the effectiveness of the sixth-line treatment and beyond would follow that of the fifth-line. To be consistent with cycle length, all rates *r* were converted into 28-day probabilities using p = 1 – *e*^[ln(1–r)/t]*4^ for *t* weeks of follow-up^24^.

Efficacy parameters for the esketamine arm were referenced from the Phase III results reported in TRANSFORM-1/2 and SUSTAIN-1 trials. Risk ratios for clinical remission (RR = 1.43, 95% CI: 1.04–1.97) and response (RR = 1.35, 95% CI: 1.14–1.61) were obtained through a random-effects meta-analysis of TRANSFORM-1 and TRANSFORM-2^11,25^. On the other hand, relapse hazard ratios were derived from the long-term SUSTAIN-1 trial^12^. The estimated hazard ratios were 0.49 (95% CI: 0.29–0.84) for patients in remission and 0.30 (95% CI: 0.16–0.55) for those in response, both in combination with background oral ADs. For other real-world treatment options, we selected evidence based on the evidence pyramid (prioritising synopsis, meta-analyses, and then randomised controlled trials), as well as recency and relevance to the current study. We extracted data on remission, response, and relapse risks from two Cochrane systematic reviews for combination therapy, augmentation, and psychotherapy^26,27^, and from seven meta-analyses for psychotherapy, rTMS, and ECT^28-34^.

- 1. **Costs of Healthcare Resource Utilisation and Treatment**

Treatment costs were derived from three primary sources: internal data, published meta-analyses^35-42^ and list of private service charges from Hospital Authority^43^. For treatments with multiple dosing regimens or heterogeneous trial designs, per-cycle treatment costs were estimated using a structured weighting and pooling approach to ensure consistency with the underlying clinical evidence. First, within each randomised controlled trial, costs associated with different dose groups were weighted according to the proportion of patients receiving each regimen: ${Weighted cost}_{trial}= \sum_{i} (\frac{n_{i}}{\sum n_{i}}\times{Cost}_{i})$, where $n_{i}$ denotes the sample size of dose group i. Second, trial-specific weighted costs were pooled across trials using meta- analysis weights aligned with the corresponding efficacy synthesis: ${Pooled\mathrm{cost}}_{cycle}= \sum_{j} (w_{j}\times{Weighted cost}_{trial, j})$, where $w_{j}$represents the relative weight of trial j in the meta-analysis. Healthcare resource utilisation (HRU) costs — including specialist outpatient clinic (SOPC), inpatient psychiatric hospital, and procedure-related services — were based on unit costs listed from Hospital Authority for non-eligible persons^44^. Details of the retrospective cohort analysis used to estimate background care costs are provided in **Fig A in S1 Appendix**. Costs were modeled at the component level, including medication costs, outpatient service costs, inpatient and emergency healthcare utilization, and treatment-related procedural costs. This structure allows individual cost components to be re-parameterized to reflect local unit prices in different healthcare systems.

- 1. **Healthcare Visits Frequency and Treatment Duration**

Healthcare visit frequency and treatment durations for each intervention directly followed those specified in the protocols of the referenced studies during derivation of clinical parameters, treatment guidelines and expert opinions to reflect real-world practice. Esketamine was modelled over a six-cycle duration, incorporating both induction and maintenance phases, based on the dosing schedules from TRANSFORM-1 and TRANSFORM-2 trials^11,25^ and prescribing guidance^45^. Combination and augmentation therapies were assumed to continue throughout the 5-year time horizon, while psychcotherapy^46-48^, rTMS^49^, and ECT^50^ were modelled as short-course interventions delivered within the first few cycles.

- 1. **Utilities**

Given that utility data were not consistently reported in the clinical trials of the interventions included in our analysis, we sourced health-related quality-of-life (HRQoL) values from Sapin et al., as they assessed the HRQoL of patient subgroups (remitters, responders, non-responders) in the most consistent context with the health states set in our model^51^. The utilities of treatment initiation and relapse states were assumed to be the same as that for non-response.

**Table A. Model input parameters**

| **Parameters** | **Value** | **Range** | **Distribution** | **Source** |
| --- | --- | --- | --- | --- |
| ***General parameters*** | | | | |
| Discount rate | 0.03 | 0.000 to 0.050 | Beta | (Wang et al., 2024) |
| Time horizon years | 5 | - | - | - |
| Annual mortality rate (%) | - | - | - | - |
| Year 1 | 2.28 | 1.32 to 3.77 | Lognormal | (Chan VKY et al., 2024) |
| Year 2 | 1.19 | 0.76 to 1.80 | Lognormal | (Chan VKY et al., 2024) |
| Year 3 | 0.91 | 0.60 to 1.35 | Lognormal | (Chan VKY et al., 2024) |
| Year 4 | 0.76 | 0.51 to 1.11 | Lognormal | (Chan VKY et al., 2024) |
| Year 5 | 0.67 | 0.45 to 0.95 | Lognormal | (Chan VKY et al., 2024) |
| ***Effectiveness parameters**** | | | | |
| **Standard care (%)** | | | | |
| Treatment to Remission |  |  |  |  |
| Third-line | 19.8% | 17.4% to 22.3% | Beta | Corrected into 4-week probabilities according to the STAR*D trial |
| Fourth-line | 10.0% | 8.2% to 11.8% | Beta |  |
| Fifth-line or above | 7.3% | 5.7% to 8.9% | Beta |  |
| Treatment to Response |  |  |  |  |
| Third-line | 18.6% | 16.2% to 20.9% | Beta | Corrected into 4-week probabilities according to the STAR*D trial |
| Fourth-line | 10.9% | 9.0% to 12.8% | Beta |  |
| Fifth-line or above | 8.1% | 6.4% to 9.7% | Beta |  |
| Remission to Relapse |  |  |  |  |
| Third-line | 12.5% | 10.6% to 14.6% | Beta | Corrected into 4-week probabilities according to the STAR*D trial |
| Fourth-line | 12.6% | 10.4% to 14.7% | Beta |  |
| Fifth-line or above | 22.8% | 20.2% to 25.4% | Beta |  |
| Response to Relapse |  |  |  |  |
| Third-line | 28.1% | 25.3% to 30.8% | Beta | Corrected into 4-week probabilities according to the STAR*D trial |
| Fourth-line | 35.9% | 32.8% to 38.8% | Beta |  |
| Fifth-line or above | 38.0% | 35.2% to 41.1% | Beta |  |
| **Effectiveness of Esketamine + AD (ESK arm)** | | | | |
| RR (Remission) | 1.43 | 1.04 to 1.97 | Lognormal | Pooled from TRANSFORM-1/2 |
| RR (Response) | 1.35 | 1.14 to 1.61 | Lognormal | Pooled from TRANSFORM-1/2 |
| HR (Relapse after remission) | 0.49 | 0.29 to 0.84 | Lognormal | SUSTAIN-1 trial |
| HR (Relapse after response) | 0.30 | 0.16 to 0.55 | Lognormal | SUSTAIN-1 trial |
| **Effectiveness of Mianserin/Mirtazapine + AD (Combination)** | | | | |
| RR (Remission) | 1.54 | 0.82 to 2.91 | Lognormal | Pooled from Cochrane review 2019 |
| RR (Response) | 1.34 | 1.00 to 1.78 | Lognormal | Pooled from Cochrane review 2019 |
| RR (Relapse) | N/A | N/A | N/A | RWE* |
| **Effectiveness of** **Olanzapine/Cariprazine/Quetiapine/Ziprasidone/Lithium+ AD (Augmentation with antipsychotics and Lithium)** | | | | |
| RR (Remission) | 1.28 | 1.12 to 1.47 | Lognormal | Pooled from Cochrane review 2019 and (Zhou et al., 2015) |
| RR (Response) | 1.27 | 1.17 to 1.38 | Lognormal | Pooled from Cochrane review 2019 and (Zhou et al., 2015) |
| RR (Relapse) | N/A | N/A | N/A | RWE* |
| **Effectiveness of Psychotherapy (Psychotherapy)** | | | | |
| OR (Improving symptoms) | 0.79 | 0.58 to 1.09 | Lognormal | Meta-analysis (van Bronswijk)* |
| RR (Relapse) | 1.02 | 0.84 to 1.25 | Lognormal | Meta-analysis (Breedvelt, 2021) |
| **Effectiveness of CBT/DBT/IPT + AD (Psychotherapy)** | | | | |
| RR (Remission) | 1.92 | 1.46 to 2.52 | Lognormal | Cochrane review 2018 |
| RR (Response) | 1.80 | 1.20 to 2.69 | Lognormal | Cochrane review 2018 |
| RR (Relapse) | 0.85 | 0.74 to 0.97 | Lognormal | Meta-analysis (Breedvelt, 2021) |
| **Effectiveness of unilateral rTMS+ AD (Neuromodulation)** | | | | |
| RR (Remission) | 1.83 | 0.94 to 3.55 | Lognormal | Meta-analysis (Sehatzadeh, 2019) |
| RR (Response) | 2.76 | 1.26 to 6.02 | Lognormal | Meta-analysis (Sehatzadeh, 2019) |
| RR (Relapse after remission) | 0.81 | 0.59 to 1.11 | Lognormal | Meta-analysis (Matsuda et al., 2023) |
| RR (Relapse after response) | 0.66 | 0.45 to 0.96 | Lognormal | Meta-analysis (Matsuda et al., 2023) |
| **Effectiveness of ECT + AD (Somatic Therapy)** | | | | |
| RR (Remission) | 3.74 | 2.25 to 6.22 | Lognormal | Meta-analysis (Zhou et al., 2014) |
| RR (Response) | 1.82 | 1.55 to 2.14 | Lognormal | Meta-analysis (Song, G.-M, 2015) |
| RR (Relapse) | 0.65 | 0.45 to 0.93 | Lognormal | Meta-analysis (Dar, H., 2023) |
| ***Cost parameters*** | | | | |
| **Healthcare resource use – Psychiatric-related outpatient visits and outreaches (HKD)** | | | | |
| Third-line | 1,165 | 1,103 to 1,251 | Gamma | Computed from CDARS ref. cohort |
| Fourth-line | 1,341 | 1,249 to 1,445 | Gamma | Computed from CDARS ref. cohort |
| Fifth-line or above | 1,686 | 1,557 to 1,825 | Gamma | Computed from CDARS ref. cohort |
| **Healthcare resource use – Psychiatric ward (HKD)** | | | | |
| Third-line | 405 | 226 to 772 | Gamma | Computed from CDARS ref. cohort |
| Fourth-line | 405 | 233 to 740 | Gamma | Computed from CDARS ref. cohort |
| Fifth-line or above | 572 | 233 to 1335 | Gamma | Computed from CDARS ref. cohort |
| **Esketamine (HKD)** | | | | |
| Treatment cost per session (3 doses) | HKD 3,912 | - | Gamma | Hong Kong Hospital Authority* |
| Psychiatric day hospital per attendance | HKD 1,260 | - | Gamma | Hong Kong Hospital Authority* |
| Nursery monitoring cost (2 hours) | HKD 335 | - | - | Hong Kong Hospital Authority* |
| Intranasal administration cost | HKD 100 | - | - | Hong Kong Hospital Authority* |
| Healthcare visits per cycle | | | | |
| Induction phase | Twice a week | - | Gamma | TRANSFORM-1/2 |
| Maintenance phase | Once a week | - | Gamma | TRANSFORM-1/2 |
| Treatment Duration cycle | | | | |
| Induction phase | 4 weeks | - | - | (Janssen Pharmaceuticals, 2024) |
| Maintenance phase | 20 weeks | - | - | (Janssen Pharmaceuticals, 2024) |
| **Combination (HKD)** | | | | |
| Weighted cost per month | HKD 25 | - | Gamma | Hong Kong Hospital Authority*; weighted using meta-analysis-derived efficacy weights |
| SOPC cost per visit | HKD1,190 | - | Gamma | Hong Kong Hospital Authority* |
| SOPC visit frequency | Once per month | - | - | Expert opinion* |
| Treatment Duration | 5 years | - | - | Expert opinion* |
| **Augmentation (HKD)** | | | | |
| Weighted cost per cycle | HKD 109 | - | Gamma | Hong Kong Hospital Authority*; weighted using meta-analysis-derived efficacy weights |
| SOPC cost per visit | HKD 1,190 | - | Gamma | Hong Kong Hospital Authority* |
| SOPC visit frequency | Once a month | - | - | Expert opinion* |
| Treatment Duration | 5 years | - | - | Expert opinion* |
| **Psychotherapies (HKD)** | | | | |
| Treatment cost (clinical psychologist consultation) per session | HKD3,150 | HKD2,520 - 3,780 | Gamma | Hong Kong Hospital Authority* |
| Treatment frequency | Once a week | - | - | (PsychDB, 2024; Peprah & Argáez, 2017; Cleveland Clinic, 2023) |
| Treatment Duration | 16 weeks | - | - | (PsychDB, 2024; Peprah & Argáez, 2017; Cleveland Clinic, 2023) |
| **rTMS (HKD)** | | | | |
| Treatment cost per session | HKD 3,000 | HKD 2,400 - 3,600 | Gamma | Hong Kong Hospital Authority* |
| SOPC cost per visit | HKD 1,190 | - | Gamma | Hong Kong Hospital Authority* |
| Treatment frequency | daily | - | - | (McClintock et al., 2018)​ |
| Treatment Duration | 6 weeks | - | - | (McClintock et al., 2018)​ |
| **ECT (HKD)** | | | | |
| Treatment cost per session | HKD 9,720 | HKD 4,860 – 14,580 | Gamma | Hong Kong Hospital Authority* |
| Psychiatric hospitals day admission | HKD 2,340 | - | Gamma | Hong Kong Hospital Authority* |
| Treatment frequency | 6 times per month | - | - | (Royal Australian and New Zealand College of Psychiatrists [RANZCP], 2019) |
| Treatment Duration | 8 weeks | - | - | (Royal Australian and New Zealand College of Psychiatrists [RANZCP], 2019) |
| *Utility weights* | | | | |
| Remission | 0.85 | 0.83 to 0.87 | Beta | Sapin et al. (2004) |
| Response | 0.72 | 0.69 to 0.75 | Beta | Sapin et al. (2004) |
| Non-response | 0.58 | 0.55 to 0.61 | Beta | Sapin et al. (2004) |
| Relapse | 0.58 | 0.55 to 0.61 | Beta | Sapin et al. (2004) |
| Treatment initiation | 0.58 | 0.55 to 0.61 | Beta | Sapin et al. (2004) |

**Notes:** One model cycle corresponds to 4 weeks. 1. Effectiveness parameters*: All effectiveness parameters represent treatment effects compared with AD monotherapy. 2. RWE*: No relevant meta-analyses or RCTs reporting added benefit of combination therapy over AD monotherapy in relapse prevention. Assumed same relapse rate as in STAR*D, which includes augmentation/combination strategies in later treatment steps. 3. Expert opinion*: Parameters labelled as “expert opinion” were informed by consensus discussions among the study co-authors, including a consultant psychiatrist, a clinical pharmacist, and a health economist, to reflect routine clinical practice in the Hong Kong public healthcare setting. 4. Hong Kong Hospital Authority*: Cost inputs labelled as “Hong Kong Hospital Authority” were derived from official Hospital Authority charge schedules and internal administrative data applicable to the 2025 price year. 5. Abbreviations: AD, Antidepressant；AP, Antipsychotic；CBT, Cognitive Behavioural Therapy；CDARS, Clinical Data Analysis and Reporting System；DBT, Dialectical Behavioural Therapy；ECT, Electroconvulsive Therapy；ESK: Esketamine；HR, Hazard Ratio；HKD: Hong Kong Dollars；IPT, Interpersonal Psychotherapy；OR, Odds Ratio；RANZCP, Royal Australian and New Zealand College of Psychiatrists；RCT, Randomised Controlled Trial；RR, Relative Risk；RWE, Real-world Evidence；SOPC, Specialist Outpatient Clinic；STAR*D, Sequenced Treatment Alternatives to Relieve Depression；rTMS, Repetitive Transcranial Magnetic Stimulation.

**Table B. Scenario analysis of per patient cost, QALYs and ICER in each comparative arm**

**Panel a. Five-year time horizon**

| **Treatment Strategy** | **Cost** | **QALYs** | **Incremental Cost (USD, vs. Ref)** | **Incremental QALYs (vs. Ref)** | **ICER (USD/QALY,vs. Ref)** |
| --- | --- | --- | --- | --- | --- |
| AUG (Ref) | 16,185 | 2.895 | Ref | Ref | - |
| ESK S8 | 18,935 | 2.950 | 2,750 | 0.055 | 50,000 |
| ESK S6 | 22,583 | 2.950 | 6,398 | 0.055 | 116,327 |
| ESK S3 | 24,612 | 2.950 | 8,427 | 0.055 | 153,218 |
| ESK S1 | 26,171 | 2.950 | 9,986 | 0.055 | 181,564 |
| ESK S4 | 26,181 | 2.950 | 9,996 | 0.055 | 181,745 |
| ESK S5 | 28,915 | 2.923 | 12,730 | 0.028 | 454,643 |
| ESK Base-case | 29,061 | 2.950 | 12,876 | 0.055 | 234,109 |
| ESK S2 | 35,898 | 3.163 | 19,713 | 0.268 | 73,556 |

**Panel b. Twenty-year time horizon**

| **Treatment Strategy** | **Cost** | **QALYs** | **Incremental Cost (USD, vs. Ref)** | **Incremental QALYs (vs. Ref)** | **ICER (USD/QALY,vs. Ref)** |
| --- | --- | --- | --- | --- | --- |
| AUG (Ref) | 50,607 | 8.994 | Ref | Ref | - |
| COM | 50,586 | 9.001 | -21 | 0.007 | Dominant* |
| PSY alone | 53,960 | 8.978 | 3,353 | -0.016 | Dominated* |
| PSY+AD | 55,978 | 9.024 | 5,371 | 0.030 | 179,033 |
| ESK S7 | 63,473 | 9.050 | 12,866 | 0.056 | 229,750 |
| rTMS+AD | 65,030 | 9.034 | 14,423 | 0.040 | 360,575 |
| ECT+AD | 80,890 | 9.103 | 30,283 | 0.109 | 227,826 |

**Notes:** 1. The base-case analysis evaluated esketamine plus antidepressant therapy (ESK + AD) under standard clinical practice assumptions. In addition to the base case, a series of prespecified scenario analyses were conducted to explore the robustness of the results to alternative clinical, costing, and methodological assumptions: **(1)ESK Scenario 1 (S1):** The esketamine maintenance dosing frequency was reduced to once every two weeks after Week 9; (2)**ESK Scenario 2 (S2):** The treatment cycle length was extended from 4 to 8 weeks, reflecting a longer clinical follow-up interval; **(3)ESK Scenario 3 (S3):** Monitoring and injection costs were applied in place of specialist outpatient clinic (SOPC) and psychiatric day hospital costs for the esketamine arm; **(4)ESK Scenario 4 (S4):** The esketamine dose per administration was reduced from three units (84 mg) to two units (56 mg); **(5)ESK Scenario 5 (S5):** A 3.5% annual discount rate was applied to both costs and health outcomes, in accordance with UK NICE reference-case guidance; **(6)ESK Scenario 6 (S6):** A 75% reduction in the esketamine acquisition price was assumed to reflect potential future price negotiations or generic entry; **(7)ESK Scenario 7 (S7):** A lifetime time horizon (20 years) was adopted to evaluate the impact of truncating the base-case analysis at 5 years.**(8) ESK Scenario 8 (S8):** Estimate the total esketamine arm cost required for esketamine to achieve cost-effectiveness at a WTP threshold of USD 50,000 per QALY, assuming unchanged effectiveness and linear cost scaling. 2. Dominant*: a strategy is associated with lower costs and higher effectiveness (QALYs) compared with the comparator. 3. Dominated*: a strategy is associated with higher costs and lower or equal effectiveness. Dominated strategies were not considered cost-effective and were therefore not assigned an ICER versus the next non-dominated strategy. 4. Abbreviations: Ref, Reference Comparator; AUG, Augmentation therapy (antidepressant combined with antipsychotic/lithium); COM, Combination therapy (antidepressant combined with antidepressant); PSY alone, Psychotherapy alone; PSY+AD, Psychotherapy combined with antidepressant; ESK+AD, Esketamine combined with antidepressant; rTMS+AD, Repetitive transcranial magnetic stimulation combined with antidepressant; ECT+AD, Electroconvulsive therapy combined with antidepressant.

**Fig A. Schema of retrospective cohort analysis for costs of healthcare resource utilization.** Blue dots represent the initiation of first-line treatment following a depression diagnosis. Orange dots indicate third-line and subsequent treatments corresponding to TRD that met criteria for adequate treatment duration. Red arrows denote periods over which healthcare resource utilisation and costs were accumulated. Black triangles indicate death. Abbreviations: DX, Diagnosis; IP, Inpatient; OP, Outpatient; TX, Treatment.


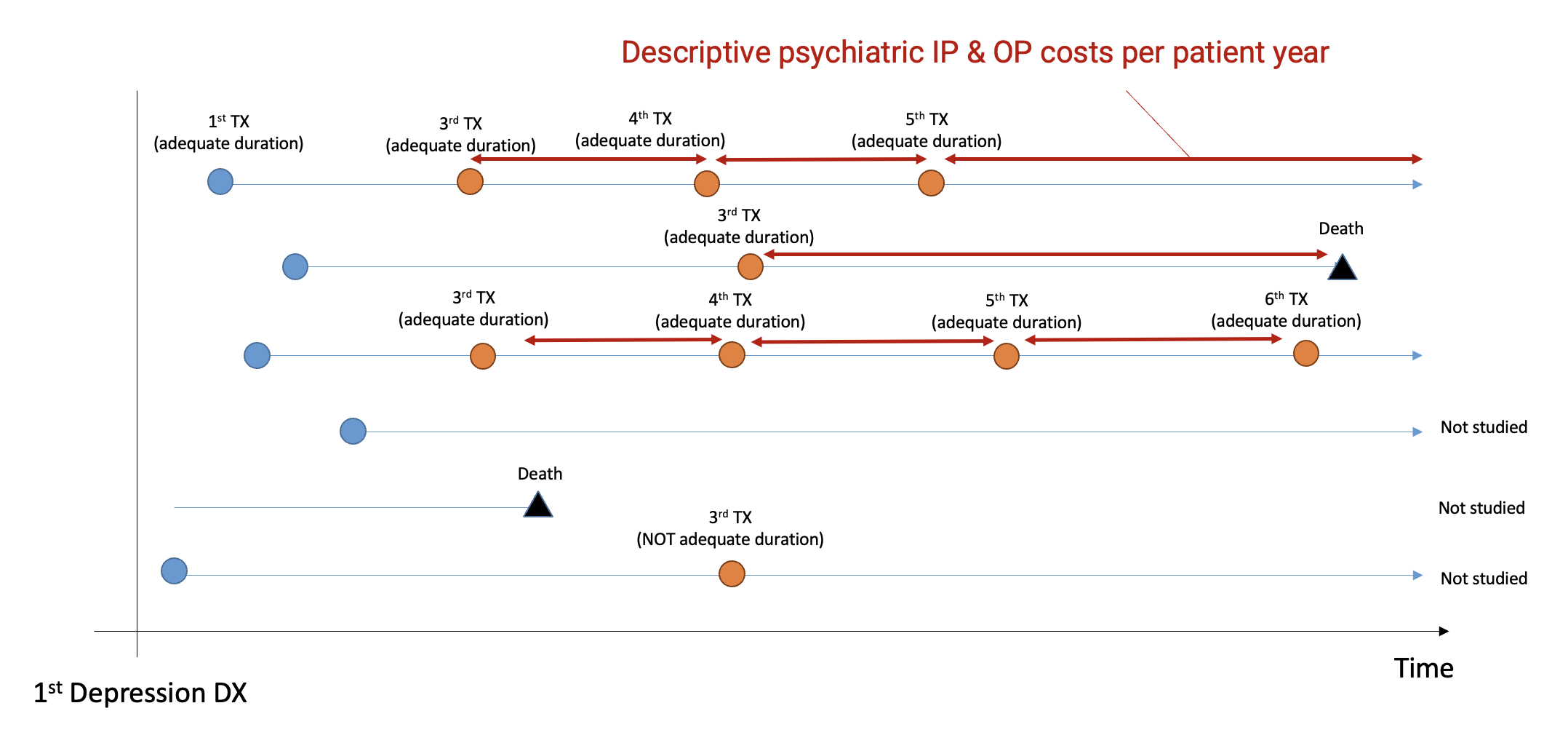


Fig B. Deterministic sensitivity analysis (DSA) results for the ICER of the esketamine (ESK) arm across key model parameters. In Panels A–E, ESK was the comparator against each respective treatment strategy; In Panel F (ECT), the reference group was set to ESK. Abbreviations: RR: relative risk indicating the effect of a treatment strategy on transition probabilities between health states; Prob: probability of transitioning between states following a treatment line; AUG, augmentation therapy (antidepressant + antipsychotic/lithium); COM, combination therapy (antidepressant + antidepressant); PSY alone, psychotherapy alone; PSY+AD, psychotherapy combined with antidepressant; ESK+AD, esketamine combined with antidepressant; rTMS+AD, repetitive transcranial magnetic stimulation combined with antidepressant; ECT+AD, electroconvulsive therapy combined with antidepressant.


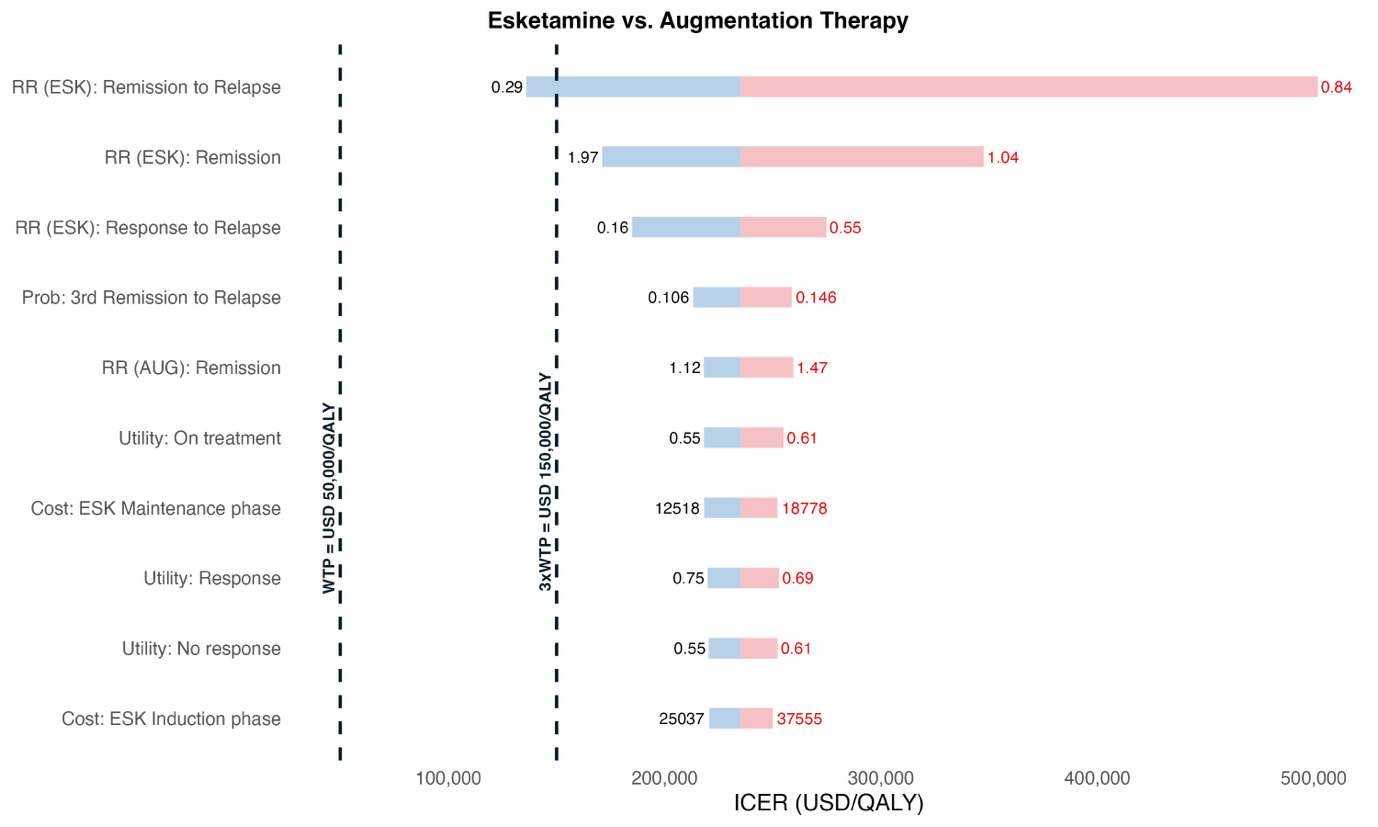


1. Esketamine vs. Augmentation Therapy


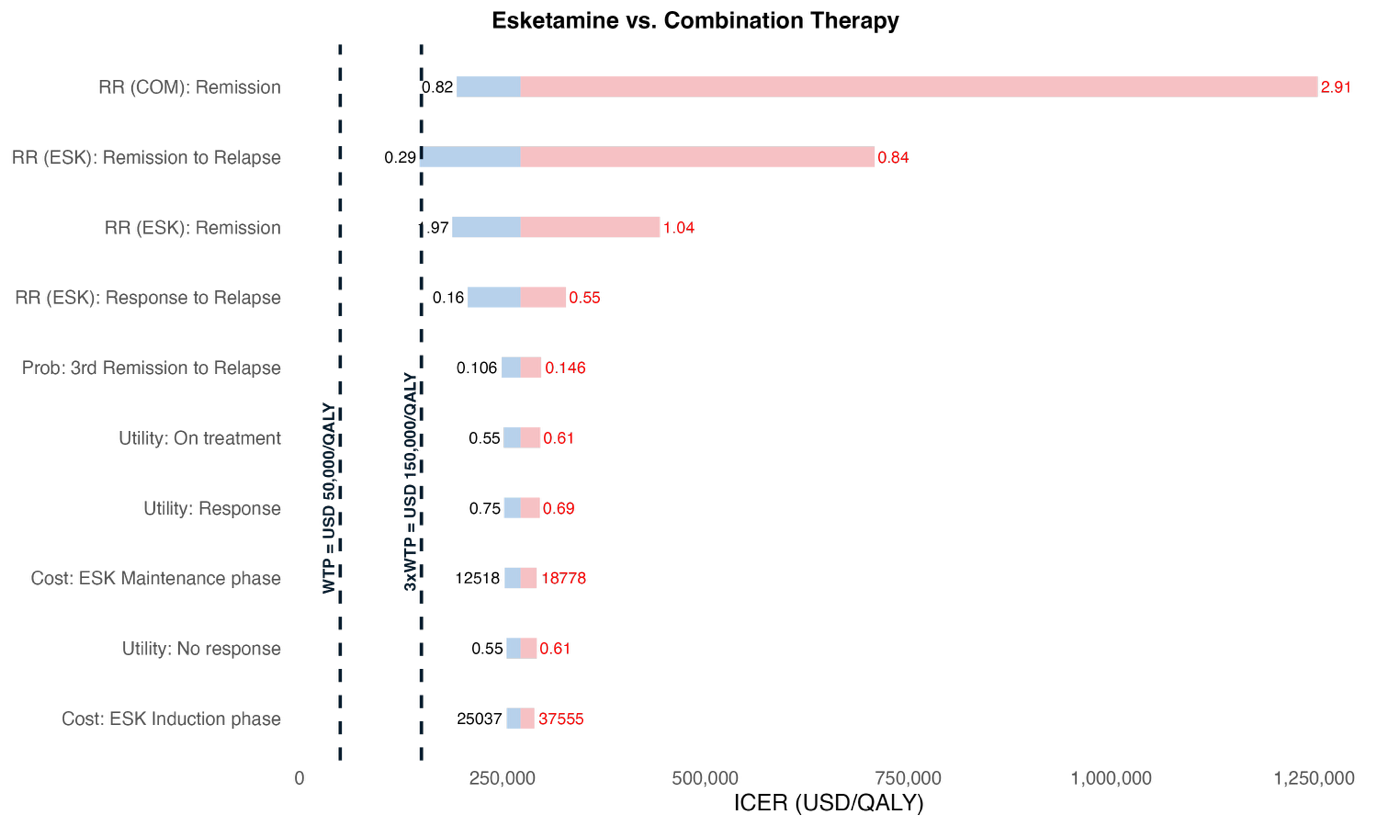


1. Esketamine vs. Combination Therapy


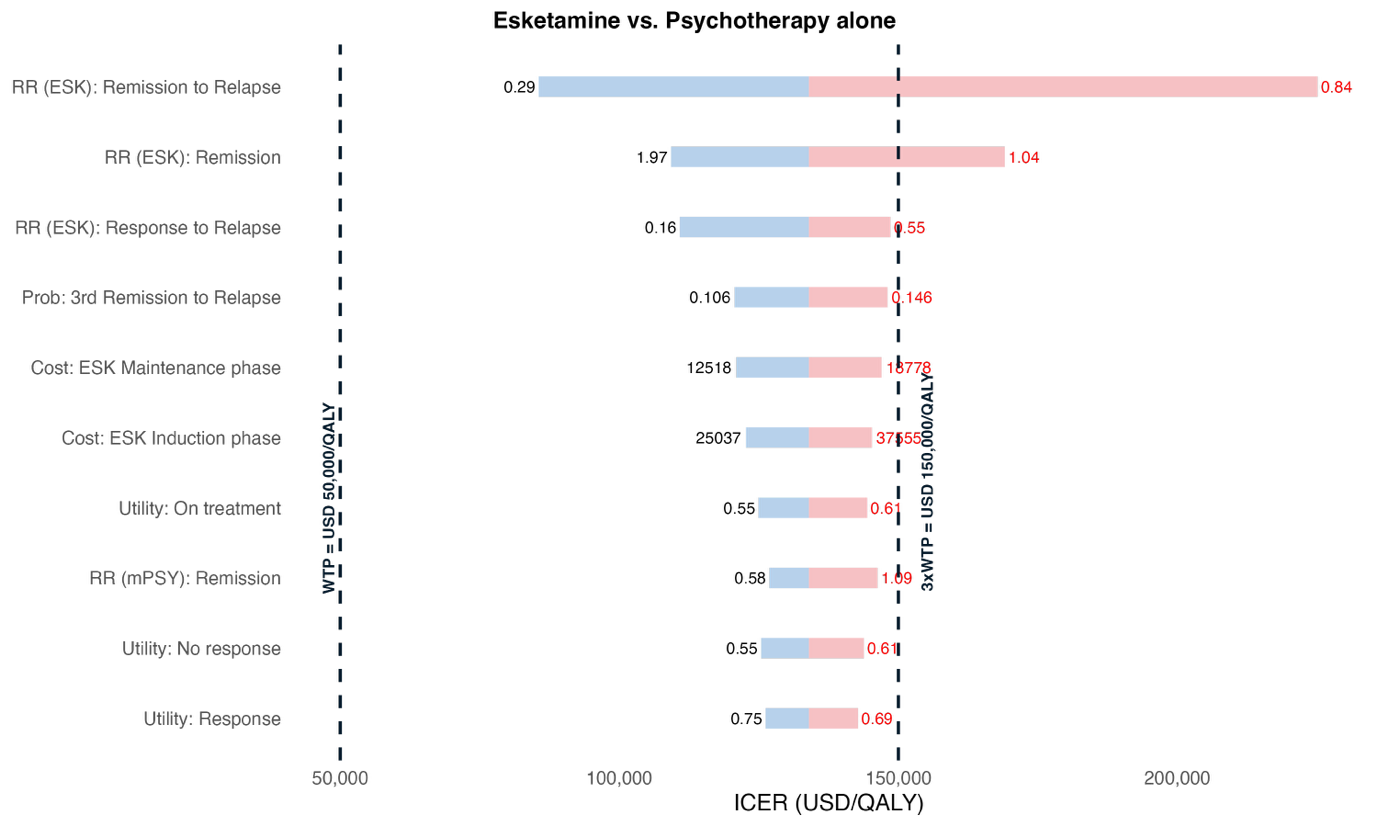


1. Esketamine vs. Psychotherapy alone


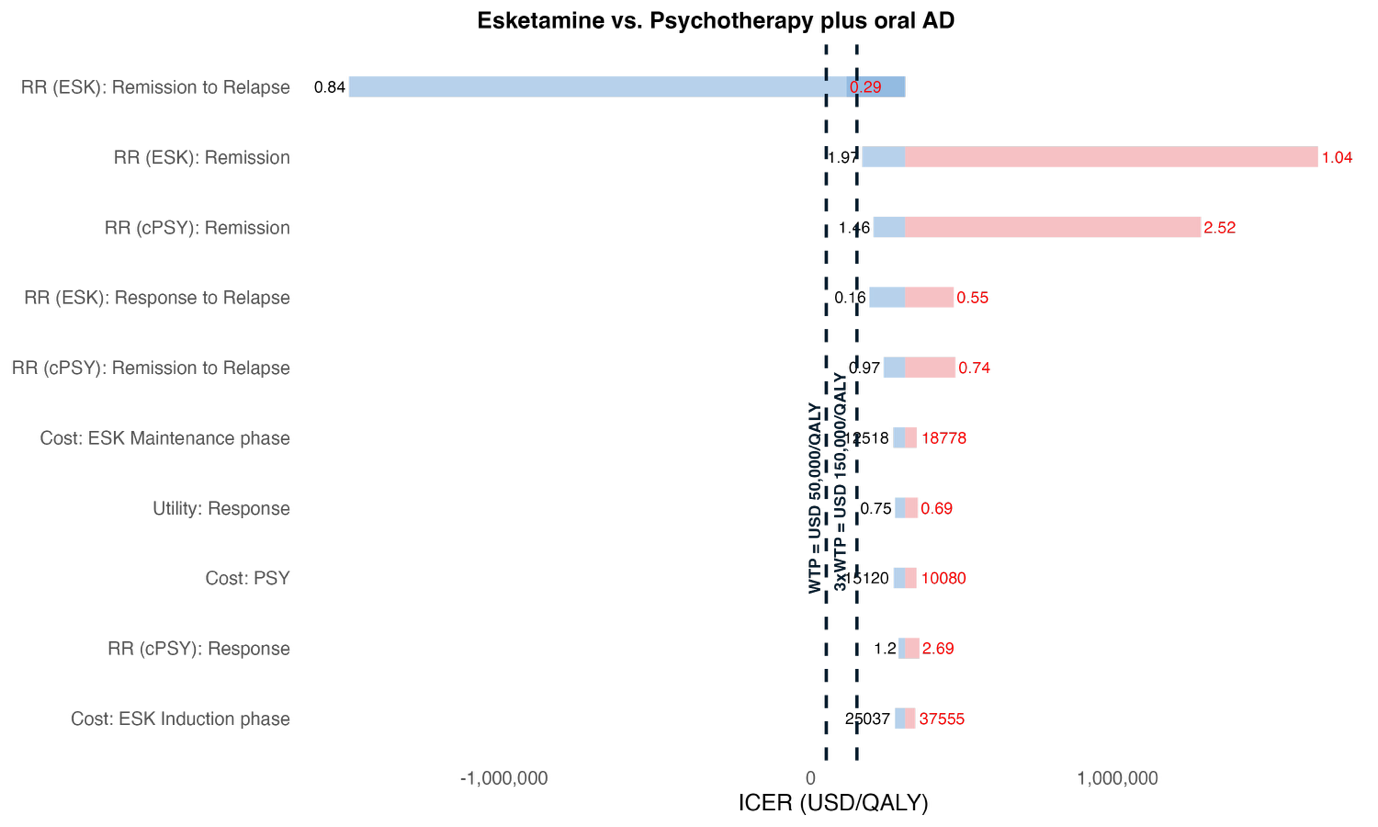


1. Esketamine vs. Psychotherapy + ADs


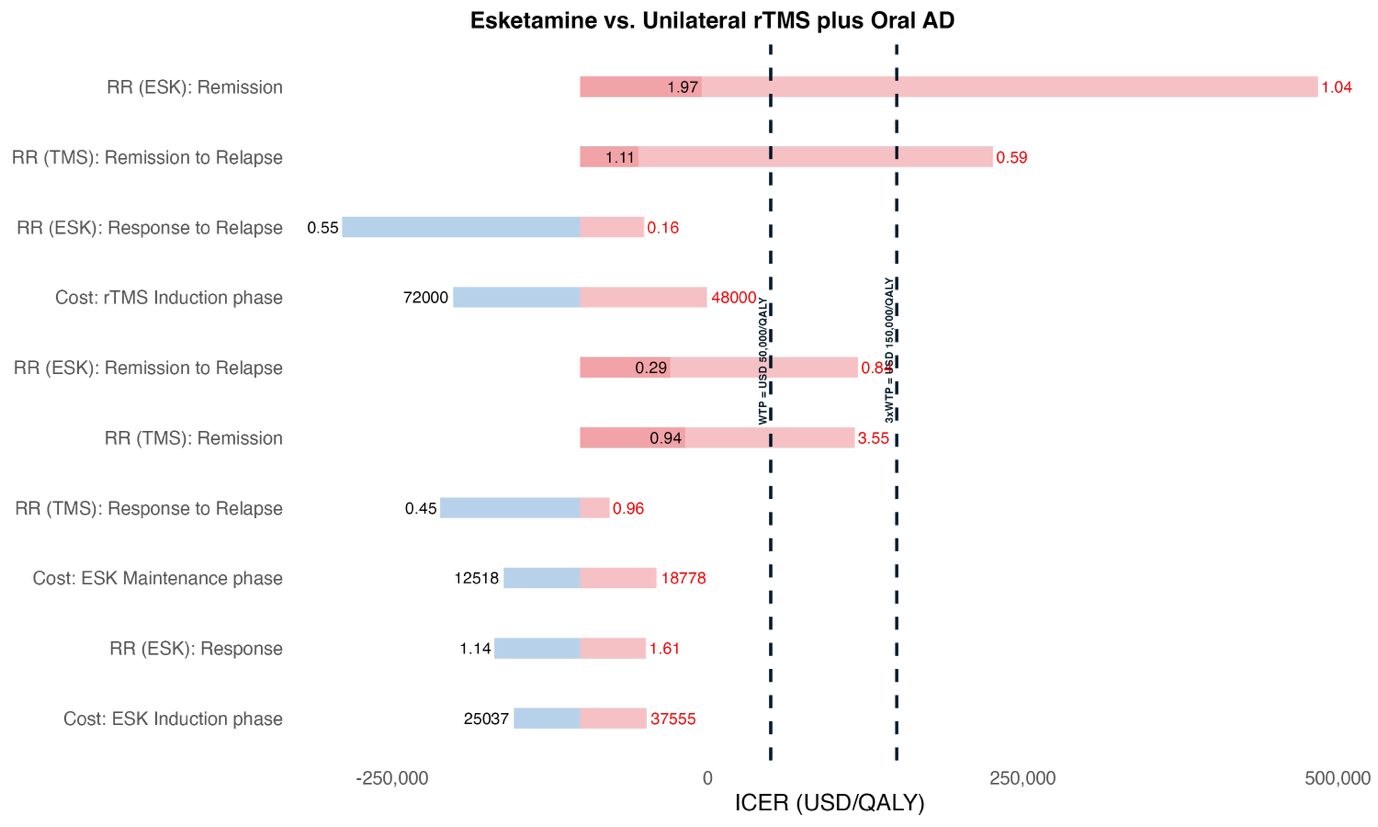


1. Esketamine vs. rTMS + ADs


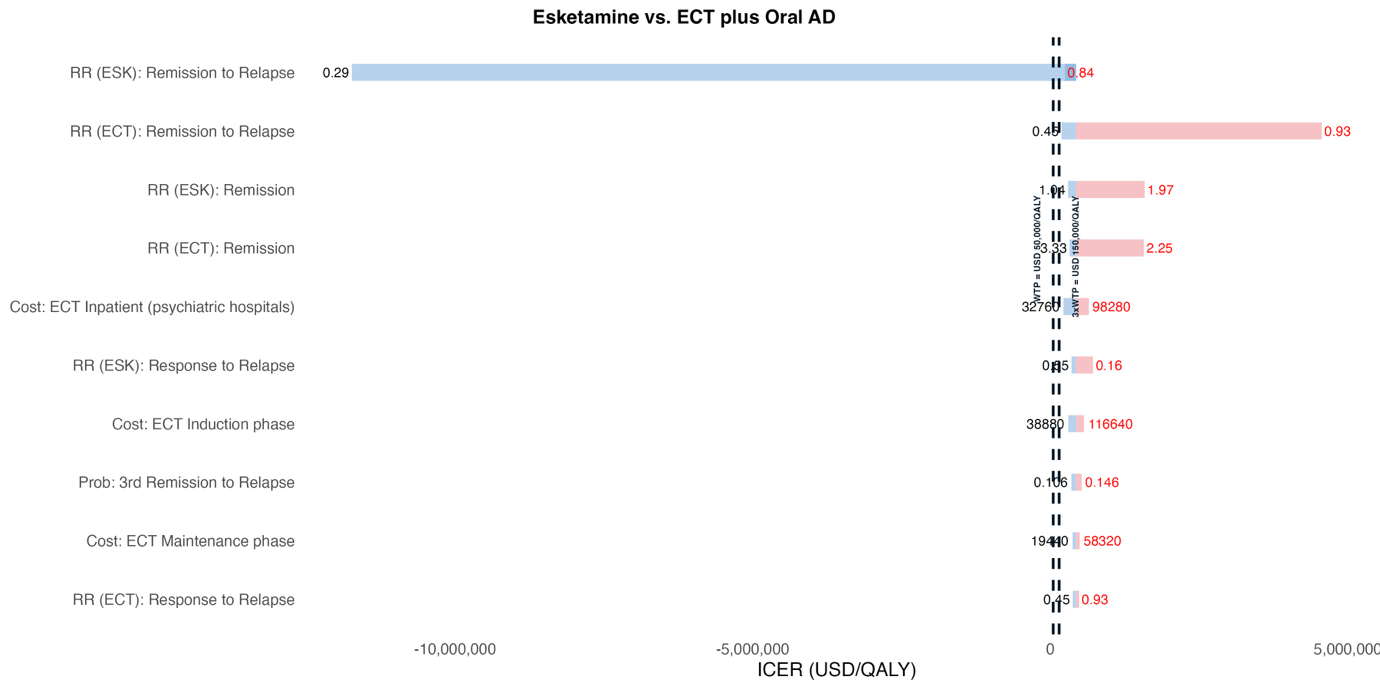


1. Esketamine vs. Electroconvulsive Therapy + ADs
