# Supplementary material for: Cost-effectiveness of esketamine versus alternative treatment strategies for treatment-resistant depression in Hong Kong: A multi-armed modeling study
Source: PLoS Med. 2026 Apr 16;23(4):e1005047. doi: 10.1371/journal.pmed.1005047 (PMC13120699; doi:10.1371/journal.pmed.1005047)
Supplement: S2 Appendix — Husereau D, Drummond M, Augustovski F, de Bekker-Grob E, Briggs AH, Carswell C, Caulley L, Chaiyakunapruk N, Greenberg D, Loder E, Mauskopf J, Mullins CD, Petrou S, Pwu RF, Staniszewska S; CHEERS 2022 ISPOR Good Research Practices Task Force. Consolidated Health Economic Evaluation Reporting Standards 2022 (CHEERS 2022) Statement: updated Reporting Guidance for Health Economic Evaluations. BMJ. 2022;376:e067975. The checklist is Open Access distributed in accordance with the terms of the Creative Commons Attribution (CC BY 4.0) license, http://creativecommons.org/licenses/by/4.0/. (DOCX) [file pmed.1005047.s002.docx]

**CHEERS 2022 Checklist**

|  | **Item** | **Guidance for Reporting** | **Reported in section** |
| --- | --- | --- | --- |
| **TITLE** | | |  |
| ^Title^ | 1 | Identify the study as an economic evaluation and specify the  interventions being compared. | Title: *Reported in Title (“Cost-effectiveness of Esketamine versus Alternative Treatment Strategies for Treatment-resistant Depression in Hong Kong: A Multi-armed Modelling Study”).* |
| **ABSTRACT** | | |  |
| Abstract | 2 | Provide a structured summary that highlights context, key methods, results and alternative analyses. | Abstract, all paragraphs |
| **INTRODUCTION** | | |  |
| Background and objectives | 3 | Give the context for the study, the study question and its practical relevance for decision making in policy or practice. | Section 1. INTRODUCTION, paras 1–4 (burden, TRD definition, esketamine, prior CEAs), and para 5 (study objective) |
| **METHODS** | | |  |
| Health economic analysis plan | 4 | Indicate whether a health economic analysis plan was developed and where available. | Code availability |
| Study population | 5 | Describe characteristics of the study population (such as age range, demographics, socioeconomic, or clinical characteristics). | Section 2.4 Model Inputs, paras 1 |
| Setting and location | 6 | Provide relevant contextual information that may influence findings. | Section 2.1 Data Sources; Section 2.5.1 Base-case Analysis, para 1 (public healthcare payer) |
| Comparators | 7 | Describe the interventions or strategies being compared and why chosen. | Section 2.2 Interventions; Abstract, Methods and Findings |
| Perspective | 8 | State the perspective(s) adopted by the study and why chosen. | Section 2.5.1 Base-case Analysis, para 1 |
| Time horizon | 9 | State the time horizon for the study and why appropriate. | Section 2.5.1, para 1 |
| Discount rate | 10 | Report the discount rate(s) and reason chosen. | Section 2.5.1, para 1 |
| Selection of outcomes | 11 | Describe what outcomes were used as the measure(s) of benefit(s) and harm(s). | Abstract, Methods and Findings; Section 2.3 Model Description; Section 2.5.1, para 1 |
| Measurement of outcomes | 12 | Describe how outcomes used to capture benefit(s) and harm(s) were measured. | Section 2.3; Section 2.4 |
| Valuation of outcomes | 13 | Describe the population and methods used to measure and value outcomes. | Section 2.4, last para (utilities from Sapin et al.) |
| Measurement and valuation of resources and costs | 14 | Describe how costs were valued. | Section 2.4; Section 2.5.2, scenarios about costing; references to HA fee schedules |
| Currency, price date, and conversion | 15 | Report the dates of the estimated resource quantities and unit costs, plus the currency and year of conversion. | Section 2.5.1 |
| Rationale and description of model | 16 | If modelling is used, describe in detail and why used. Report if the model is publicly available and where it can be accessed. | Section 2.3; Fig 1; Section 2.5.1, para 1 |
| Analytics and assumptions | 17 | Describe any methods for analysing or statistically transforming data, any extrapolation methods, and approaches for validating any model used. | Section 2.4, paras 1 (rate to probability conversion formula); Section 2.5.1–2.5.2; description of scenario structures |
| Characterizing heterogeneity | 18 | Describe any methods used for estimating how the results of the study vary for sub-groups. | NA; analysis conducted for overall adult TRD cohort. No subgroup-specific cost-effectiveness estimates were generated |
| Characterizing distributional effects | 19 | Describe how impacts are distributed across different individuals or adjustments made to reflect priority populations. | NA; no explicit distributional or equity weighting was applied. |
| Characterizing uncertainty | 20 | Describe methods to characterize any sources of uncertainty in the analysis. | Section 2.5.2 Scenario and Sensitivity Analyses, all paras; Section 3.2–3.3; Figs 3–4 |
| Approach to engagement with patients and others affected by the study | 21 | Describe any approaches to engage patients or service recipients, the general public, communities, or stakeholders (e.g., clinicians or payers) in the design of the study. | NA; no formal patient or public involvement in design or conduct of the economic evaluation. |
| **RESULTS** | | |  |
| Study parameters | 22 | Report all analytic inputs (e.g., values, ranges, references) including uncertainty or distributional assumptions. | Section 2.4 (for inputs, with Table A in S1 Appendix) |
| Summary of main results | 23 | Report the mean values for the main categories of costs and outcomes of interest and summarise them in the most appropriate overall measure. | Abstract, Methods and Findings + Conclusions; Section 3.1 Base-case Analysis; Fig 2; Table 1 |
| Effect of uncertainty | 24 | Describe how uncertainty about analytic judgments, inputs, or projections affect findings. Report the effect of choice of discount rate and time horizon, if applicable. | Section 3.2 Scenario Analysis; Section 3.3 Sensitivity Analyses; Figs 3–4; Fig B and Table B in S1 Appendix |
| Effect of engagement with patients and others affected by the study | 25 | Report on any difference patient/service recipient, general public, community, or stakeholder involvement made to the approach or findings of the study | NA; no formal stakeholder or patient engagement was conducted, hence no effect on design or results |
| **DISCUSSION** | | |  |
| Study findings, limitations, generalizability, and current knowledge | 26 | Report key findings, limitations, ethical or equity considerations not captured, and how these could impact patients, policy, or practice. | Section 4. DISCUSSION, all paragraphs |
| **OTHER RELEVANT INFORMATION** | | | |
| Source of funding | 27 | Describe how the study was funded and any role of the funder in the identification, design, conduct, and reporting of the analysis | Funding, all paragraphs |
| Conflicts of interest | 28 | Report authors conflicts of interest according to journal or  International Committee of Medical Journal Editors requirements. | Conflicts of interest, all paragraphs |

Husereau D, Drummond M, Augustovski F, de Bekker-Grob E, Briggs AH, Carswell C, Caulley L, Chaiyakunapruk N, Greenberg D, Loder E, Mauskopf J, Mullins CD, Petrou S, Pwu RF, Staniszewska S; CHEERS 2022 ISPOR Good Research Practices Task Force. Consolidated Health Economic Evaluation Reporting Standards 2022 (CHEERS 2022) Statement: Updated Reporting Guidance for Health Economic Evaluations. BMJ. 2022;376:e067975.

The checklist is Open Access distributed in accordance with the terms of the Creative Commons Attribution (CC BY 4.0) license, which permits others to distribute, remix, adapt and build upon this work, for commercial use, provided the original work is properly cited. See: http://creativecommons.org/licenses/by/4.0/.
