# Supplementary material for: Cost-effectiveness of esketamine versus alternative treatment strategies for treatment-resistant depression in Hong Kong: A multi-armed modeling study
Source: PLoS Med. 2026 Apr 16;23(4):e1005047. doi: 10.1371/journal.pmed.1005047 (PMC13120699; doi:10.1371/journal.pmed.1005047)
Supplement: Attachment — Submitted filename: cea_response_letter_FINAL.docx [file pmed.1005047.s005.docx]

March 24, 2026

Till Winfried Bärnighausen,

Editor-in-chief

PLOS Medicine

Evangelia Fourli,

Associate Editor

PLOS Medicine

Dear Dr. Till Bärnighausen and Dr. Evangelia Fourli,

Thank you very much for your consideration of our manuscript. We are grateful to the editor and reviewers for their constructive and insightful comments, which have helped us improve the clarity and quality of our work.

We have carefully revised the manuscript in response to all comments. In particular, we have addressed all general editorial and formatting requirements, including updates to the abstract structure, Author Summary, statistical reporting, and figure formatting, in accordance with PLOS Medicine guidelines. All requested changes have been incorporated throughout the revised manuscript. Below, we provide a detailed point-by-point response to each comment.

Thank you again for your time and consideration of our manuscript.

Yours sincerely,

**Dr. Shirley Xue Li**, PhD MPhil

Assistant Professor

Department of Medicine & Department of Pharmacology and Pharmacy

LKS Faculty of Medicine

The University of Hong Kong

PB306, 3/F, Professional Block, Queen Mary Hospital

102 Pok Fu Lam Road, Hong Kong

Email: [sxueli@hku.hk](mailto:sxueli@hku.hk)

**Professor Sandra Sau Man Chan**

Department of Psychiatry

Faculty of Medicine

The Chinese University of Hong Kong,

Email: [schan@cuhk.edu.hk](mailto:schan@cuhk.edu.hk)

**Editor’s Comment to the Authors**

**Comment 1:**

You mentioned that the GitHub link will be available before publication, which is a requirement to publish your work. Please include a Zenodo link or DOI as well.

**Author Response:**

We have now archived the code repository in a publicly accessible Zenodo repository and included the corresponding DOI (10.5281/zenodo.19187012) in the Code Availability statement to ensure long-term accessibility and reproducibility.

These changes have been made in the revised Code availability section:

*“The analytical code used to develop the economic model and perform the analyses is publicly available on GitHub (https://github.com/scan2030/scan2030-WP-3_TRD_ESK_CEA) and archived on Zenodo (https://doi.org/10.5281/zenodo.19187012).”*

(Page 31, lines 2-5, Code availability)

**Comment 2:**

While you indicate that your study uses anonymized data, please include a clear statement regarding informed consent.

**Author Response:**

We have now added a statement in the Methods section clarifying that the requirement for informed consent was waived due to the use of fully anonymized data.

These changes have been made in the revised Methods section:

*“The study protocol was approved by the Institutional Review Board of the University of Hong Kong / Hospital Authority Hong Kong West Cluster (HKU/HA HKW IRB) (Approval No.: UW 20-218). The requirement for informed consent was waived due to the use of fully anonymized data. All model inputs, assumptions, and results were independently cross-checked by two independent researchers (YL and VC) to ensure quality, accuracy and transparency.”*

(Page 8, lines 5-11, Methods)

**Reviewers’ Comment to the Authors**

**Reviewer 1**
All of Reviewer 1's comments have now been addressed.

**Author Response:**

We thank the reviewer for their positive feedback and for their constructive comments, which have helped improve the clarity and rigor of the manuscript.

**Reviewer 2**

We sincerely thank the reviewer for the very positive and thoughtful comments. We are grateful for the reviewer’s recognition of the clinical usefulness of our study and for the valuable suggestions to further strengthen the manuscript.

**Minor Comment 1:**

While I understand that the perspective of the article is "currently in Hong Kong", I still would have found the inclusion of IV Ketamine as a treatment option interesting. Perhaps it should be a treatment option in Hong Kong? Or perhaps it should not? Either way, it is extensively used in the US and it might have been possible to model "potential cost" in Hong Kong based on available data.

Suggestion: No change is needed. It is not something that I consider vital to this article. However, if IV ketamine is not added to this article, I would be very interested in reading about the results if you, in future works, added IV Ketamine to your current model to see where a "potential use" of IV Ketamine would fit in regard to cost-effectiveness in Hong Kong.

**Author Response:**

We thank the reviewer for this insightful and constructive suggestion, which we believe adds important perspective to our study. We fully agree that intravenous (IV) ketamine represents a clinically relevant comparator and an important direction for future research.

We did consider the inclusion of IV ketamine during the study design phase. However, it was not incorporated into the current analysis for several reasons. First, in the Hong Kong public healthcare setting, IV ketamine is not routinely used as part of a standardized treatment pathway for TRD, and there is limited availability of locally relevant data on its utilization, costs, and real-world effectiveness. Although it would be possible to approximate cost inputs using data from other healthcare systems, such assumptions would introduce substantial uncertainty and reduce the context-specific validity of the model. Second, IV ketamine is typically used off-label in the treatment of depression and is associated with considerable heterogeneity in dosing protocols, monitoring requirements, and service delivery models, which complicates its parameterization within a robust and standardized economic framework.^1^

Importantly, emerging economic evidence suggests that the cost-effectiveness of IV ketamine is highly sensitive to the analytical perspective. For example, recent trial-based economic evaluations reported that ketamine may be cost-effective, or even dominant, under a healthcare system perspective, but not necessarily under a patient or societal perspective.^2-3^ This discrepancy largely reflects differences in cost structure across different perspectives: while the acquisition cost of ketamine is relatively low, the overall cost of treatment is driven by administration, monitoring, and productivity losses associated with repeated treatment sessions, which are more fully captured under patient and societal perspectives. In contrast, esketamine is associated with substantially higher drug acquisition costs but benefits from standardized delivery protocols and regulatory approval and more established reimbursement pathways. As a result, the relative cost-effectiveness of these treatments may vary considerably depending on the analytical perspective and local healthcare system characteristics.

Therefore, IV ketamine may represent a potentially cost-effective comparator under certain healthcare system perspectives. We agree with the reviewer that evaluating its role within a Hong Kong-specific context would be highly valuable. Accordingly, we have added statements in the Discussion to highlight the potential role of IV ketamine, including its distinct cost structure and implications for generalizability, as well as its importance as an avenue for future research, particularly in healthcare systems where its use is more established in clinical practice.

These changes have been made in the revised Discussion section:

*“In healthcare systems with lower labor costs, the relative cost-effectiveness of these interventions may differ substantially, even if clinical effectiveness remains unchanged. This is particularly relevant for labor-intensive or service-dependent interventions. For example, treatments such as intravenous ketamine (IV ketamine) have cost structures that are highly dependent on administration, monitoring, and service delivery requirements, making their economic value sensitive to local implementation practices. The component-level cost reporting in this study enables context-specific re-estimation and supports adaptation of the model to alternative healthcare settings.”*

(Page 21, lines 11-18, Discussion)

*“Lastly, IV ketamine was not included as a comparator in this study. Although it is increasingly used in some healthcare settings, it is not routinely incorporated into standardized treatment pathways for TRD within the Hong Kong public healthcare system, and there is limited availability of locally relevant data on its utilization, costs, and real-world effectiveness. While it would be possible to approximate cost inputs using data from other settings, such assumptions would introduce substantial uncertainty and reduce the contextual validity of the model. In addition, its off-label use and heterogeneity in administration protocols present further challenges for robust economic modelling. Given its distinct cost structure — characterized by low drug acquisition cost but relatively higher administration and service-related costs — its cost-effectiveness is likely to be context-dependent and may vary substantially across healthcare systems. Future research incorporating IV ketamine as a comparator would be valuable in healthcare systems where its use is more established in clinical practice, enabling more context-specific and policy-relevant economic evaluations.”*

(Page 25, lines 18-23; Page 26, lines 1-8, Discussion)

**Comment 2:**

I think that this article highlights an important and often overlooked aspect of treatment options in clinical practice, namely relative cost-effectiveness. If all things are equal, cost effectiveness might inform choice of treatment. In fact, one could argue that third-forth-fifth-line treatment recommendations might be (at least to a degree) guided by cost-effectiveness.

Suggestion: No change is needed. But I think a comment regarding how findings such as the ones in your article may help form more comprehensive guidelines beyond "first-second-third"-line might be warranted. Perhaps a sentence added to conclusions?

**Author Response:**

We thank the reviewer for this valuable suggestion. We agree that cost-effectiveness evidence may help inform more comprehensive treatment sequencing and resource allocation decisions beyond conventional line-of-therapy recommendations. In response, we have added text in the Discussion and Conclusion to highlight how findings from comparative economic evaluations such as ours may contribute to the development of more comprehensive and context-sensitive clinical guidance for later-line treatment strategies in treatment-resistant depression.

These changes have been made in the revised Discussion and Conclusion sections:

*“More broadly, findings from comparative cost-effectiveness analyses may help inform more comprehensive and context-sensitive treatment sequencing strategies for TRD, beyond conventional line-of-therapy frameworks. Such evidence may support clinical decision-making and resource allocation in later-line treatment settings.”*

(Page 24, lines 1-5, Discussion)

*“Importantly, our findings highlight that cost-effectiveness evidence may help inform more context-sensitive treatment sequencing strategies beyond conventional line-of-therapy frameworks. These insights may support reimbursement decision-making, pricing negotiations, and the design of more efficient care delivery models for TRD management.”*

(Page 26, lines 15-18, Conclusion)

**Reviewer 3**

Thank you for adequately addressing my concerns. I have no further comments.

**Author Response:**

We sincerely appreciate the reviewer’s supportive comments and are pleased that our revisions have adequately addressed their concerns.

**References**

1. Swainson, J., McGirr, A., Blier, P., Brietzke, E., Richard-Devantoy, S., Ravindran, N., Blier, J., Beaulieu, S., Frey, B. N., Kennedy, S. H., McIntyre, R. S., Milev, R. V., Parikh, S. V., Schaffer, A., Taylor, V. H., Tourjman, V., van Ameringen, M., Yatham, L. N., Ravindran, A. V., & Lam, R. W. (2021). The Canadian Network for Mood and Anxiety Treatments (CANMAT) Task Force Recommendations for the Use of Racemic Ketamine in Adults with Major Depressive Disorder: Recommandations Du Groupe De Travail Du Réseau Canadien Pour Les Traitements De L'humeur Et De L'anxiété (Canmat) Concernant L'utilisation De La Kétamine Racémique Chez Les Adultes Souffrant De Trouble Dépressif Majeur. Canadian journal of psychiatry. Revue canadienne de psychiatrie, 66(2), 113–125. <https://doi.org/10.1177/0706743720970860>
2. Chatterton, M. L., Perez, J. K., Thai, T., Faller, J., Loo, C. K., Glozier, N., Barton, D., Baune, B. T., Mills, N. T., Fitzgerald, P. B., Glue, P., Sarma, S., Hadzi-Pavlovic, D., Dong, V., Martin, D., Mitchell, P. B., Berk, M., Carter, G., Hackett, M., Hood, S., … Mihalopoulos, C. (2025). Economic evaluation of subcutaneous ketamine injections for treatment resistant depression: A randomised, double-blind, active-controlled trial - The KADS study. Journal of affective disorders, 387, 119502. <https://doi.org/10.1016/j.jad.2025.119502>
3. Brendle, M., Robison, R., & Malone, D. C. (2022). Cost-effectiveness of esketamine nasal spray compared to intravenous ketamine for patients with treatment-resistant depression in the US utilizing clinical trial efficacy and real-world effectiveness estimates. Journal of affective disorders, 319, 388–396. https://doi.org/10.1016/j.jad.2022.09.083
