# Supplementary material for: Cost-effectiveness of esketamine versus alternative treatment strategies for treatment-resistant depression in Hong Kong: A multi-armed modeling study
Source: PLoS Med. 2026 Apr 16;23(4):e1005047. doi: 10.1371/journal.pmed.1005047 (PMC13120699; doi:10.1371/journal.pmed.1005047)
Supplement: Attachment — Submitted filename: R3_cea_response_letter_final.docx [file pmed.1005047.s007.docx]

March 27, 2026

Till Winfried Bärnighausen,

Editor-in-chief

PLOS Medicine

Evangelia Fourli,

Associate Editor

PLOS Medicine

Dear Dr. Till Bärnighausen and Dr. Evangelia Fourli,

Thank you very much for your continued consideration of our manuscript, entitled “Cost-effectiveness of Esketamine versus Alternative Treatment Strategies for Treatment-resistant Depression in Hong Kong: A Multi-armed Modelling Study” (PMEDICINE-D-25-03549R3).

We are grateful for the editor’s careful review and helpful comments. We have carefully revised the manuscript and supplementary materials accordingly. In particular, we have addressed the remaining editorial and formatting issues raised, including reference placement, Author Summary formatting, wording revisions, and manuscript structure, in accordance with PLOS Medicine style and reporting requirements.

Below, we provide a point-by-point response to each comment.

Thank you again for your time and consideration of our manuscript.

Yours sincerely,

**Dr. Shirley Xue Li**, PhD MPhil

Assistant Professor

Department of Medicine & Department of Pharmacology and Pharmacy

LKS Faculty of Medicine

The University of Hong Kong

PB306, 3/F, Professional Block, Queen Mary Hospital

102 Pok Fu Lam Road, Hong Kong

Email: [sxueli@hku.hk](mailto:sxueli@hku.hk)

**Professor Sandra Sau Man Chan**

Department of Psychiatry

Faculty of Medicine

The Chinese University of Hong Kong,

Email: [schan@cuhk.edu.hk](mailto:schan@cuhk.edu.hk)

**Editor’s Comment to the Author**

**Comment 1:**

Both in the main text and supplementary information, you have references inserted after the punctuation. Can you please revise both files and make sure that references are included before punctuation (including commas, full stops, semi colons etc).

**Author Response:**

Thank you for this comment. We have carefully reviewed both the main manuscript and supplementary information and revised the placement of all in-text references so that citations now consistently appear before punctuation, in accordance with PLOS Medicine style requirements. These changes have been made throughout the revised manuscript and supplementary files.

**Comment 2:**

Please, replace arrows with bullet points in the authors summary.

**Author Response:**

Thank you for this suggestion. We have revised the Author Summary by replacing arrows with standard bullet points, in line with journal formatting preferences.

This change has been made in the revised Author Summary section:

*“AUTHOR SUMMARY*

*Why was this study done?*

*•	Treatment-resistant depression (TRD) is common and places a substantial burden on patients and healthcare systems.*

*•	Esketamine is a novel and fast-acting treatment, but its economic value compared with treatments used in routine clinical practice remains unclear.*

*•	Previous economic studies mainly compared esketamine with less realistic alternatives, rather than with the broader range of treatment strategies commonly used in clinical care.*

*What did the researchers do and find?*

*•	We developed a cost-effectiveness model to compare esketamine with six commonly used third-line treatment strategies for adults with TRD in Hong Kong over a five-year period.*

*•	We found that esketamine was more economically favourable than neurostimulation therapies such as repetitive transcranial magnetic stimulation (rTMS) and electroconvulsive therapy (ECT), but was not cost-effective compared with commonly used strategies such as combination therapy, augmentation therapy, or psychotherapy-based treatments at standard decision thresholds.*

*•	Among all strategies, combination therapy was the most cost-effective option, and the main findings remained broadly similar when we tested different assumptions in the model.*

*What do these findings mean?*

*•	Although esketamine offers clinical benefits, its high cost currently limits its economic value compared with other widely used treatments, and improving its cost-effectiveness may require price reductions or more efficient service delivery.*

*•	These findings may help inform clinical guidelines and healthcare policy decisions by supporting more cost-effective treatment sequencing strategies.*

*•	This study is limited by its use of a modelling approach and reliance on data from multiple sources rather than direct patient comparisons.”*

(Page 4; Page 5 lines 1-7, Author Summary)

**Comment 3:**

Line 10: I suggest removing independently as independent reviews should convey the meaning.

**Author Response:**

Thank you for this suggestion. We have removed the word “independently” from the relevant sentence to improve conciseness and avoid redundancy.

This change has been made in the revised manuscript:

*“All model inputs, assumptions, and results were cross-checked by two independent researchers (YL and VC) to ensure quality, accuracy and transparency.”*

(Page 8, line 12-14, Methods)

**Comment 4:**

Please remove conclusions as an independent text section; it should be included in the discussion. Generally, the discussion should have no subsections.

**Author Response:**

Thank you for this helpful comment. We have revised the manuscript structure accordingly by removing the standalone “Conclusions” subsection and integrating the concluding content into the final part of the Discussion section. We have also ensured that the Discussion is presented without subsections, in line with journal style.

These changes have been made in the revised Discussion section

*“Overall, this study provides a comprehensive real-world–informed economic evaluation of esketamine compared with multiple third-line treatment strategies for TRD. While esketamine was not cost-effective compared with most alternatives under the current WTP threshold, it may offer relative economic advantages over certain neurostimulation treatments such as rTMS and ECT. Importantly, our findings highlight that cost-effectiveness evidence may help inform more context-sensitive treatment sequencing strategies beyond conventional line-of-therapy frameworks. These insights may support reimbursement decision-making, pricing negotiations, and the design of more efficient care delivery models for TRD management.”*

(Pages 26, line 13-20, Discussion)

**Comment 5:**

In the abstract, line 23, you state: “comparing seven third-line strategies”. Can I please confirm that this is phrased correctly based on your analysis? In the text it is always mentioned as esketamine vs 6 treatments, so I am wondering if this could be misread. If you believe that this is correct, please disregard this comment.

**Author Response:**

Thank you for this important clarification. We agree that the original wording could potentially be misinterpreted. To improve clarity and better reflect the structure of our analysis, we have revised the abstract wording from “comparing seven third-line strategies” to “comparing esketamine with six alternative third-line treatment strategies”.

This change has been made in the revised Abstract:

*“The model compared esketamine plus AD with six alternative third-line treatment strategies: combination therapy (AD plus AD), augmentation therapy (AD plus antipsychotic or lithium), psychotherapy alone, psychotherapy plus AD, repetitive transcranial magnetic stimulation (rTMS) plus AD, and electroconvulsive therapy (ECT) plus AD. Primary outcomes were quality-adjusted life-years (QALYs) and incremental cost-effectiveness ratios (ICERs) under a US$50,000/QALY willingness-to-pay (WTP) threshold.”*

(Pages 3, line 1-6, Methods and Findings, Abstract)
